# Olfactory Cues from Plants Infected by Powdery Mildew Guide Foraging by a Mycophagous Ladybird Beetle

**DOI:** 10.1371/journal.pone.0023799

**Published:** 2011-08-18

**Authors:** Jun Tabata, Consuelo M. De Moraes, Mark C. Mescher

**Affiliations:** 1 Department of Entomology, Pennsylvania State University, University Park, Pennsylvania, United States of America; 2 Biodiversity Division, National Institute for Agro-Environmental Sciences, Tsukuba, Ibaraki Prefecture, Japan; University of Wisconsin-Milwaukee, United States of America

## Abstract

Powdery mildews (Erysiphales) are economically important plant pathogens that attack many agricultural crops. Conventional management strategies involving fungicide application face challenges, including the evolution of resistance and concerns over impacts on non-target organisms, that call for investigation of more sustainable alternatives. Mycophagous ladybird beetles (Coleoptera: Coccinellidae) feed on powdery mildew and have considerable potential as biological control agents; however, the foraging ecology and behavior of these beetles is not well understood. Here we document the olfactory cues presented by squash plants (*Cucurbita moschata*) infected by powdery mildew (*Podosphaera* sp.) and the behavioral responses of twenty-spotted ladybird beetles (*Psyllobora vigintimaculata*) to these cues. Volatile analyses through gas chromatography revealed a number of volatile compounds characteristic of infected plants, including 3-octanol and its analogues 1-octen-3-ol and 3-octanone. These compounds are typical “moldy” odorants previously reported in volatiles collected from other fungi. In addition, infected plants exhibited elevated emissions of several compounds also observed in collections from healthy leaves, including linalool and benzyl alcohol, which are reported to have anti-fungal properties. In Y-tube choice assays, *P. vigintimaculata* beetles displayed a significant preference for the odors of infected plants compared to those of healthy plants. Moreover, beetles exhibited strong attraction to one individual compound, 1-octen-3-ol, which was the most abundant of the characteristic fungal compounds identified. These results enhance our understanding of the olfactory cues that guide foraging by mycophagous insects and may facilitate the development of integrated disease-management strategies informed by an understanding of underlying ecological mechanisms.

## Introduction

Powdery mildews (Ascomycota: Erysiphales) are obligately biotrophic fungi that attack a wide range of plant species and infect many different plant structures [1]; consequently, these fungi are among the most economically important plant pathogens in agricultural ecosystems [2]. Management of powdery mildew typically entails regular fungicide application, but conventional control methods can be costly and, moreover, face growing concerns about the emergence of resistance and potential impacts on non-target organisms and human health [3]. Such concerns favor the development of potential alternative control strategies, including biological control by arthropods or microbes (e.g. [4]–[10]). However, the effective implementation of such strategies will require a sophisticated understanding of underlying ecological mechanisms [11].

Mycophagous ladybird beetles in the tribe Halyziini (Coleoptera: Coccinellidae) are potentially attractive agents for the biological control of powdery mildew [12], [13], but our understanding of the trophic ecology of these beetles is currently limited. In particular we know almost nothing about the foraging cues that guide beetles to mildew-infected plants. To begin to address this gap in our knowledge, the current study documents the olfactory cues emitted by butternut squash plants, *Cucurbita moschata*, infected by powdery mildew, *Podosphaera* sp., and the behavioral responses to these cues exhibited by the twenty-spotted ladybird beetle, *Psyllobora vigintimaculata* (Say). Plant-associated volatiles are key foraging cues both for plant feeding insects and for the natural enemies of plant antagonists (e.g. [14], [15]), and the effective implementation of biological control agents and other ecological control strategies often hinges on understanding and manipulating complex ecological interactions that are frequently mediated by chemistry [16], [17].

All members of the tribe Halyziini feed primarily on powdery mildews in both their larval and adult stages, though they sometimes also utilize sooty molds or pollen as alternative food sources [18]. Like the fungi that they feed upon, the Halyziini exhibit a cosmopolitan distribution, and it appears that at least one species of mycophagous ladybird is present wherever powdery mildews commonly occur [13]. The twenty-spotted ladybird is a relatively small beetle distributed throughout North America and commonly found in agricultural and horticultural systems [19]. It has been suggested that this insect might usefully be employed alongside other management strategies to facilitate effective control of powdery mildew [12], as an individual larva can clear all visible powdery-mildew hyphae and conidia from an estimated leaf area of 6.3 cm^2^ [20].

While little is currently known about the foraging behavior of mycophagous ladybirds, entomophagous ladybirds are known to utilize olfactory cues when foraging [21]. For example, both adults and larvae of *Adalia bipunctata* are attracted to volatiles associated with their aphid prey, and the aphid alarm pheromone (*E*)-β-farnesene is an effective attractant for this species [22]. Another predatory ladybird, *Coccinella septempunctata*, was found to respond positively to the odors of aphid-infested plants—and to aphid-induced volatiles emitted by previously infested plants [23]—as well as to (*E*)-β-farnesene [24]. And *Hippodamia convergens* individuals exhibited stronger attraction to the combination of odors deriving directly from aphids and plant volatiles induced by aphid feeding than to either class of cues presented alone [25]. As the Halyziini are believed to be derived from a sub-group of the tribe Coccinellini, which comprises primarily aphidophagous species [26], it is reasonable to suspect that mycophagous species are similarly responsive to olfactory cues when foraging for food, though this has not previously been demonstrated.

In order to explore the potential use of olfactory cues by mycophagous ladybirds foraging for powdery mildew, we collected volatiles emitted from butternut squash plants infected by powdery mildew greenhouse conditions and analyzed them by gas-chromatography (GC) and mass spectrometry, comparing the observed chemical profiles to those of intact plants. Then we investigated behavioral responses of twenty-spotted ladybird adults to the odors of infected and uninfected plants using a Y-tube behavioral bioassay. Finally, we used similar methods to assess beetle responses to individual components of the mildew-associated blend. The results reported here provide new insight into the volatile chemistry of powdery mildew infections and the foraging ecology of mycophagous beetles and have potential implications for the development of novel strategies for integrated disease management.

## Results

### Volatiles emitted from squash plants infected by powdery mildew

**Figure 1:**
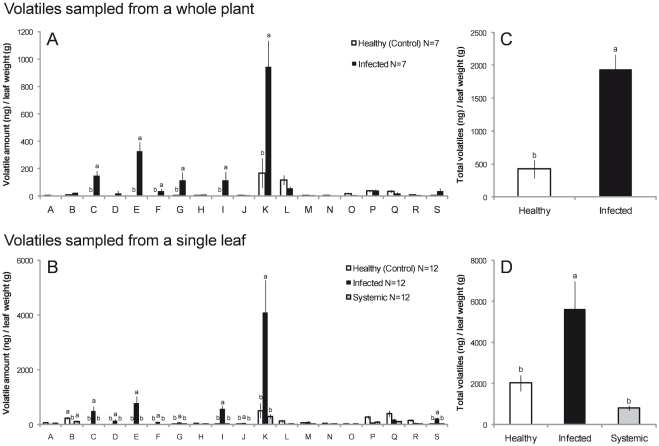
Amounts of volatiles (mean ±**SE) emitted from healthy and powdery mildew-infected plants in the greenhouse.** Panels A and B show individual compounds from whole plants and single leaves, respectively: A, (*E*)-2-hexenal; B, (*Z*)-3-hexenol; C, unknown; D, 3-octanone; E, 1-octen-3-ol; F, 3-octanol; G, benzyl alcohol; H, (*E*)-β-ocimene; I, octanol; J, nonanal; K, linanlool; L, (*E*)-4,8-dimethyl-1,3,7-nonatriene; M, ethylbenzaldehyde; N, 4-ethylbenzaldehyde; O, nonanol; P, decanal; Q, ethylacetophenone; R, ethylacetophenone; S, decanol. (The pairs of compounds, (i) M and N and (ii) Q and R, were determined to be isomers of ethylbenzaldehyde and ethylacetophenone, respectively.) Panels C and D show total volatiles. “Systemic” refers leaves without powdery mildew on infected plants. Lowercase letters indicate significant differences among treatments (*P*<0.05 by ANOVA followed by Tukey-Kramer HSD).

Our analyses of volatiles emitted from healthy and infected squash plants as well as from individual leaves identified several compounds that were characteristic of infection and observed only when powdery mildew was present (Fig. 1A,B), including 3-octanol and two of its analogues, 1-octen-3-ol and 3-octanone (3-octanol, *F*_1,12_ = 59.85, *P*<0.0001 for whole plants, *F*_2,33_ = 836.4, *P*<0.0001 for single leaves; 1-octen-3-ol, *F*_1,12_ = 602.1, *P*<0.0001 for whole plants, *F*_2,33_ = 616.7, *P*<0.0001 for single leaves; 3-octanone, *F*_1,12_ = 3.770, *P* = 0.1689 for whole plants, *F*_2,33_ = 19.76, *P*<0.0001 for single leaves). An additional, unidentified compound suggested by the mass spectrum data to be octadien-3-ol (also an analogue of 3-octanol)—exhibiting characteristic fragment ions at *m*/*z* = 126 (0.5%, M^+^), 108 (10.5), 91 (11.8), 79 (23.2), 69 (33.7), and 57 (100)—was characteristic of infected plants (*F*_1,12_ = 409.3, *P*<0.0001 for whole plants, *F*_2,33_ = 274.5, *P*<0.0001 for single leaves).

Furthermore, infected plants exhibited significantly higher emissions of several compounds that were also present in the volatile blends of healthy plants, including linalool (*F*_1,12_ = 17.82, *P* = 0.0034 for whole plants, *F*_2,33_ = 17.43, *P*<0.0001 for single leaves), benzyl alcohol (*F*_1,12_ = 26.66, *P* = 0.0004 for whole plants, *F*_2,33_ = 11.20, *P* = 0. 0.0008 for single leaves), and octanol (*F*_1,12_ = 107.0, *P*<0.0001 for whole plants, *F*_2,33_ = 74.00, *P*<0.0001 for single leaves). Decanol was also significantly elevated for single leaves (*F*_2,33_ = 25.90, *P*<0.0001), though its elevation in whole plant samples was not significant. Conversely, the emission of (*Z*)-3-hexenol was significantly decreased in single leaves infected by powdery mildew (*F*_2,33_ = 13.08, *P*<0.0001), though this difference was also not apparent in whole plant samples.

Total volatile emissions (Fig. 1C,D) were significantly increased in infected plants (*F*_1,12_ = 23.54, *P* = 0.0013) and individual leaves (*F*_2,33_ = 17.20, *P*<0.0001), although volatile emissions from individual leaves without powdery mildew on infected plants (i.e., “systemic” leaves) was lower than that of healthy leaves.

The *P* values presented above were adjusted by using the Benjamini and Hochberg method [27] to control the false discovery rate. The elevation of decanol in whole plant samples was marginally significant prior to this adjustment; the significance of all other results was robust. Because the control data were all zeros for compounds not present in the uninfected plants, these data were reanalyzed using non-parametric statistic methods (Wilcoxon/Kruskal-Wallis tests), which yielded results consistent with those of the parametric analyses presented (ANOVA).

### Behavioral responses of the twenty spotted ladybird to volatiles from powdery mildew-infected plants

**Figure 2:**
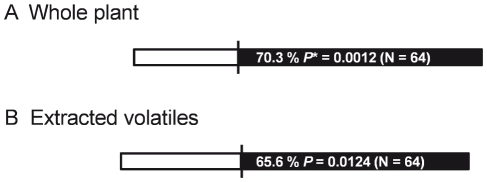
Twenty-spotted ladybird responses to natural volatiles emitted from healthy (□) and infected (▪) plants in a Y-tube behavioral assay. A: an assays using whole plants; B: an assays using volatiles collected from whole plants, eluted in solvent (CH_2_Cl_2_), and re-evaporated from a filter-paper disc. *: *P*-value of chi-square test against the expectation of random (1∶1) beetle choices.

**Figure 3:**
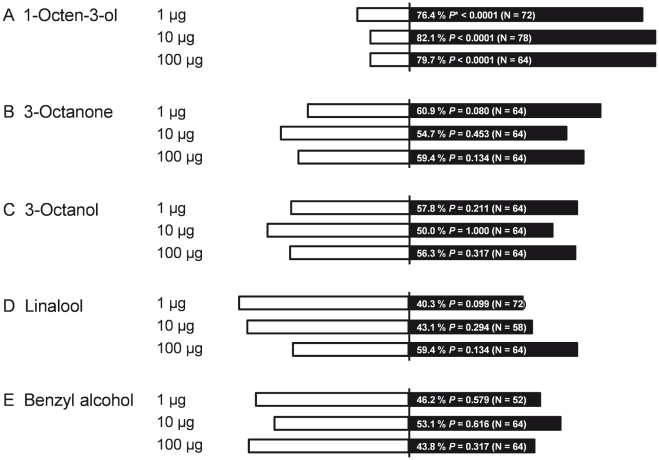
Twenty-spotted ladybird responses to synthetic volatiles (▪) versus solvent (CH_2_Cl_2_) controls (□) in a Y-tube behavioral assay. *: *P*-value of chi-square test against the expectation of random (1∶1) beetle choices.

In Y-tube bioassays beetles exhibited a significant preference for the odors of infected plants compared to those of healthy plants (Fig. 2A), and this preference persisted when the assay was repeated using extracted plant volatiles (Fig. 2B). We also tested beetle responses to individual compounds that were found to be characteristic of powdery mildew infection or were significantly elevated in infected plants (versus solvent controls; Fig. 3) and found that only 1-octen-3-ol elicited a strong behavioral response at the dosages examined (1 to 100 µg). More beetles also responded to 3-octanone than to controls across all trials (*N* = 192, *χ* ^2^ = 5.33, *P* = 0.0209), but this preference was not statistically significant for any single concentration tested.

## Discussion

Our results clearly demonstrate that the olfactory cues emitted from squash plants infected with powdery mildew are significantly different than those of healthy plants and that twenty-spotted ladybird beetles can distinguish the odors of healthy and infected plants and preferentially respond to the latter. Moreover, one volatile compound characteristic of powdery mildew infection, 1-octen-3-ol, was significantly attractive to beetles when presented in isolation. This compound and its analogues 3-octanol and 3-octanone, which were also characteristic of infection (Fig. 1), are typical “moldy” odorants commonly found in volatiles collected from fungi, including powdery mildews (e.g. [28]–[30]); thus, it is likely that these compounds are derived from the fungi themselves rather than from infected plant tissues.

These chemicals, which are known products of the oxidation and cleavage of linoleic acid [28], [31], [32], have previously been implicated in fungal attraction of insects. For example, 1-octen-3-ol was reported to attract gravid females of the fly *Megaselia halterat*a, a pest of mushroom crops [33]. Moreover, strictly fungivorous beetles (Ciidae) have been found to respond to volatile cues from wood-rotting fungi and to discriminate among fungal host species on the basis of odorants including 1-octen-3-ol [34]. Fäldt et al. [35] previously suggested that 3-octanol and its analogues might attract predators of mycophagous insects. Presumably, these apparently ubiquitous fungal odors provide useful foraging cues for mycophagous ladybirds, which can potentially feed on a broad range of powdery mildew species infecting a variety of host plants [13], [36].

We also observed elevated emissions from infected plants of several compounds that were also present in the volatile blends of healthy plants as well, including linalool, benzyl alcohol, octanol, and decanol (Fig. 1). Both linalool and benzyl alcohol have previously been suggested to have anti-fungal activity (e.g. [37]–[39]); thus, elevated emissions of these compounds may reflect the induction plant defenses against powdery mildew infection. It is also plausible that such compounds may play an indirect role in plant defense by facilitating the attraction of natural enemies of the fungus. Such a role is well established for plant volatiles induced by herbivory (e.g. [14]), and tritrophic interactions involving powdery mildew (*Uncinula necator*), riverbank grapevines (*Vitis riparia*), and mycophagous mites (*Orthotydeus lambi*) have previously been documented [40]–[41]. We observed no significant attraction of beetles to synthetic linalool and benzyl alcohol when these compounds were presented in isolation. Nevertheless, the potential contribution of plant-derived compounds to the attractiveness of the overall blend emitted by infected plants cannot be entirely discounted, as insect responses to olfactory foraging cues appear frequently to rely on complex informational characteristics of the overall volatile blend rather than on attraction to individual compounds (e.g. [42]–[44]).

While most mycophagous ladybirds accept a wide range of powdery mildew species as food sources, some exhibit host preferences or restrictions of host range [13], which may be mediated by the recognition of plant-specific cues. For example, in California *P*. *vigintimaculata* have not been reported on *Euonymus japonica* or *Eschscholtzia california* plants severely infected by *Oidium* spp. and *Erysiphe* spp., respectively, even though they commonly feed on these fungi on other plants, including *Salvia spathacea* (*Oidium*), *Zinnia elegans* (*Erysiphe*), or *Cucumis sativa* (*Erysiphe*) [13]. Similarly, an Asian mycophagous ladybird, *Illeis koebelei*, exhibits foraging preferences between different plant species associated with the same genera of powdery mildew [36].

Several previous studies have documented pathogen-induced changes in plant odors that lead to increased attraction of insect disease vectors (e.g. [45]–[48]). Fungal plant pathogens that rely on insect pollinators for the transmission of gametes or the dissemination spores often emit, or induce the emission of, olfactory cues that mimic floral odors [49]–[51]. For example, Raguso and Roy [49] analyzed the volatile emissions of pseudoflowers induced by rusts in the *Puccinia monoica* complex on several species of cruciferous plants in the mustard genus *Arabis* and found them to be composed primarily of aromatic alcohols, aldehydes, and esters—while many of the fragrant compounds emitted by fungi-induced pseudoflowers appear to be produced by the fungal parasite, at least some compounds in the blends emitted are suspected to be of host origin [50]. In contrast with these previously explored systems, powdery mildew is an air-borne pathogen believed to be transmitted from infected to uninfected plants primarily by mechanical forces, such as wind [1]; although mycophagous ladybirds can potentially transport powdery mildew conidia from one plant to another, the transmission of mildew was not found to be significantly enhanced in the presence of adult ladybirds in a growth-chamber assay [13]. Thus, the attraction of mycophagous lady beetles to the odors of infected plants is presumably not adaptive for the fungi in this system, an observation consistent with the apparent use by *P*. *vigintimaculata* of volatile cues that are broadly characteristic of many fungi and potentially of plant compounds characteristic of anti-fungal defense.

In summary, our results document characteristic olfactory cues associated with the infection of squash plants by powdery mildew, including the emission of characteristic compounds that have previously been reported from other fungi and elevated emissions of apparently plant-derived compounds that may be induced by fungal infection. On the whole volatile emissions from infected plant tissues are elevated relative to healthy tissues. The twenty-spotted ladybird beetle exhibits significant attraction to the characteristic odors of mildew-infected plants as well as attraction to one compound associated with infection, 1-octen-3-ol, which was the most abundant of the characteristic fungal compounds identified in our study. These observations provide insight into the use of olfactory foraging cues by mycophagous ladybird beetles, which have not previously been explored, and provide basic information about the chemical ecology of tritrophic plant-fungus-beetle interactions that may facilitate the development of integrated disease-management strategies informed by an understanding of underlying ecological mechanisms.

## Materials and Methods

### Plants and powdery mildew

Butternut squash *Cucurbita moschata* seeds were planted in ∼140 cm^3^ of potting soil (Pro-Mix; Premier Tech, Quebec, Canada) and grown in a greenhouse under a 14L:10D photoperiod at 23°C. Powdery mildew, *Podosphaera* sp., was collected from field-grown squash at the Penn State Rocksprings Agricultural Experiment Station in Ramblewood PA on August 15, 2010 and inoculated on *C*. *moschata* in the greenhouse: ∼1 cm^2^ of mildew (∼3.9×10^4^ conidia) were picked up using a soft paintbrush and gently transferred to a single leaf of each one-week-old plant. To prevent dispersal of spores and unintentional infection, plants were placed in trays (25.4×50.8 cm) and covered with clear plastic domes (17.8 cm in height). Three-week-old plants (inoculated 2 weeks previously) were used for volatile collections.

### Insects

Twenty-spotted ladybird beetles (*P*. *vigintimaculata*) were collected in University Park, PA, during June-October, 2010. The beetles were fed on powdery mildew (growing on squash plants inoculated as above) and maintained in the greenhouse, as follows: plastic containers (35×22.5×27 cm) with two pots of squash well-infected by powdery mildew each contained ∼20 adult beetles that were allowed to mate and lay eggs on the potted plants. Plants with eggs were transferred to trays with transparent domes, as described above. After hatching, larvae were kept on the plants until all visible mildew was consumed, then gently transferred to a new powdery mildew-infected plant using a soft brush. Once formed, pupae were placed in Petri dishes (9 cm diameter) until eclosion.

### Volatile collections

Whole plants were placed in closed chambers: 4-L glass-domes with Teflon bases (*N* = 7 per treatment). Specially designed 15 cm×17 cm square glass leaf chambers with Teflon frames were used to enclose single (intact) leaves (*N* = 12 per treatment). Charcoal-filtered air was pushed into the chamber and pulled through Super-Q (Alltech, Deerfield, IL) traps (push/pull flow rates 4.0/2.0 L per min for whole plants and 1.6/0.8 L per min for single leaves). Volatiles were collected over 5 hours during the light phase of the photoperiod. Samples were eluted from the Super-Q traps using 150 µl of CH_2_Cl_2_; and 10 µl of an internal standard (20 ng/µl nonyl acetate and 10 ng/µl octane; Sigma-Aldrich, St. Louis, MO) was added.

### Chemical analyses of volatiles

Aliquots (2 µl) of samples collected as described above were injected into an Agilent 6890 gas chromatograph (GC; Agilent Technologies, Palo Alto, CA) fitted with a flame ionization detector (FID) or an Agilent 5973 mass spectrometer (MS) in the splitless mode for 0.3 min. An apolar HP-1 column (15 m×0.25 mm I.D., 0.25 µm film thickness for GC-FID; 30 m×0.25 mm I.D., 0.25 µm film thickness for GC-MS; Agilent Technologies) was installed, and the column oven temperature was maintained at 35°C for 5 min, then raised at 3.75°C/min to 240°C, and maintained for 7 min. Temperatures of the injector, the FID, and the MS interface were kept at 250°C, respectively. Tentative identifications of compounds were made by calculation of Kovat's indices and comparison of these values with a previously compiled list of known compounds and indices and by retention time comparisons with authentic standards as well as comparison of mass spectra with a library of known compounds. Amounts of volatiles (ng) produced per dry weight of plant tissues (g) was quantified using MSD Chemstation (Agilent Technologies) by measuring peak area relative to the internal standard and dividing this value by the dry weight of a sample. The scores (*X*) were transformed to log (*X*+1) before statistical analysis (ANOVA followed by Tukey-Kramer Honestly Significant Difference tests and Wilcoxon/Kruskal-Wallis tests) using JMP (version 5.1.1, SAS Institute 2004).

### Y-tube bioassays of beetle responses to olfactory cues

The behavioral responses of adult (one to three-week-old) twenty-spotted ladybird beetles were investigated by means of two-choice tests using a Y-tube olfactometer (19×17×17 cm arm-length, 1 cm diameter, 60° Y angle), which was positioned vertically in the greenhouse. A first experiment examined the beetle responses to infected and uninfected whole plants enclosed in the 4-L glass chambers described above. Chambers containing infected and uninfected plants were connected to the ends of the Y-tube by flexible Teflon tubes. Charcoal-filtered air was pushed into each of the chambers at 2.0 L/min and pulled through the Y-tube at and 1.0 L/min. Individual beetles were placed at downwind end of the Y-tube using a soft brush and were considered to have made a choice when they arrived at upwind end of the tube. Around 10% of beetles remained at the downwind end for ≥5 min; these were deemed unresponsive and excluded from the analyses.

A second experiment used similar methods to examined beetle responses to plant volatiles extracted by CH_2_Cl_2_ using the methods described above. An aliquot (50 µl) of the extract was loaded onto a filter-paper disc and re-evaporated. Treatment and control discs (which received only solvent) were placed at the two ends of the Y-tube and air was pushed and pulled through the Y-tube at a rate of 1.0 L/min. Behavioral responses were assayed as above.

Finally, beetle responses to individual compounds characteristic of infected plants were assayed using the same methods employed for extracted plant volatiles. The following commercially acquired synthetic compounds were tested: 1-octen-3-ol (98%; Aldrich), 3-octanone (≥98%; Aldrich), 3-octanol (≥97% Sigma-Aldrich Fine Chemicals), linalool (97%; Aldrich), and benzyl alcohol (≥99%; Aldrich).
